# Statistical analysis of organelle movement using state-space models

**DOI:** 10.1186/s13007-023-01038-6

**Published:** 2023-07-05

**Authors:** Haruki Nishio, Satoyuki Hirano, Yutaka Kodama

**Affiliations:** 1 Data Science and AI Innovation Research Promotion Center, Shiga University, Shiga, 522‑8522 Japan; 2 Center for Ecological Research, Kyoto University, Shiga, 520‑2113 Japan; 3 Center for Bioscience Research and Education, Utsunomiya University, Tochigi, 321-8505 Japan; 4 Graduate School of Regional Development and Creativity, Utsunomiya University, Tochigi, 321-8505 Japan

**Keywords:** Chloroplast, Accumulation response, Movement start time, Signal transfer speed, Bayesian inference, Kalman filter

## Abstract

**Background:**

Organelle motility is essential for the correct cellular function of various eukaryotic cells. In plant cells, chloroplasts move towards the intracellular area irradiated by a weak light to maximise photosynthesis. To initiate this process, an unknown signal is transferred from the irradiated area to distant chloroplasts. Quantification of this chloroplast movement has been performed using visual estimations that are analyst-dependent and labour-intensive. Therefore, an objective and faster method is required.

**Results:**

In this study, we developed the cellssm package of R (https://github.com/hnishio/cellssm.git), which is a user-friendly tool for state-space modelling to statistically analyse the directional movement of cells or organelles. Our method showed a high accuracy in estimating the start time of chloroplast movement in the liverwort *Marchantia polymorpha* over a short period. The tool indicated that chloroplast movement accelerates during transport to the irradiated area and that signal transfer speed is uneven within a cell. We also developed a method to estimate the common dynamics among multiple chloroplasts in each cell, which clarified different characteristics among cells.

**Conclusions:**

We demonstrated that state-space modelling is a powerful method to understand organelle movement in eukaryotic cells. The cellssm package can be applied to various directional movements (both accumulation and avoidance) at cellular and subcellular levels to estimate the true transition of states behind the time-series data.

**Supplementary Information:**

The online version contains supplementary material available at 10.1186/s13007-023-01038-6.

## Background

In eukaryotic cells, organelles dynamically change their subcellular positions along cytoskeletal filaments using motor proteins to maintain correct cellular functioning [1, 2]. Among the various organelles, photosynthetic chloroplasts have been highly studied for their motility in plant cells because their intracellular positioning is important for the optimization of photosynthesis, which contributes to agricultural application (e.g., plant biomass) [3, 4]. Chloroplasts constantly change their intracellular positions by a random walk due to cytosolic streaming, and also show actin-dependent directional movement in response to environmental factors such as light in various plant species such as the fern *Adiantum capillus-veneris*, thale cress *Arabidopsis thaliana*, and liverwort *Marchantia polymorpha* [5]. This directional movement of chloroplasts optimises photosynthetic performance [3, 6]. For example, under strong light conditions, chloroplasts move away from the intracellular area irradiated by the light to reduce photodamage of photosynthetic machinery (avoidance response) [6]. In contrast, chloroplasts move towards the weak-light-irradiated area to maximise light perception (accumulation response), which promotes leaf photosynthesis and overall biomass production [3]. For the majority of plant species, the avoidance and accumulation responses can be induced by blue light, mediated by the blue-light receptor phototropin, which mainly localises at the plasma membrane [5]. In some plants, such as *A. capillus-veneris*, the accumulation response is also induced by red light which is mediated by the red-light receptor neochrome [7, 8]. In *A. thaliana*, chloroplasts move using chloroplast actin (cp-actin) filaments, which are short actin filaments that emerge from the chloroplast edge [9].

The process of inducing the accumulation response appears to be divided into three steps: (1) phototropin is activated by blue light, (2) an unknown signal is transferred from the activated phototropin to chloroplasts, and (3) after receiving the signal, the chloroplast moves using cp-actin to the area containing the activated phototropin. Even if strong-blue-light is used in the first step, the processes involved in the second and third steps still occur [5, 9].

Several methods have been developed to quantify chloroplast movement, such as the measurement of leaf transmittance, evaluation of chlorophyll fluorescence, and direct tracking of chloroplasts [10–12]. Among these methods, the direct tracking allows for an investigation of the behaviour of each chloroplast and the creation of a dataset consisting of multiple chloroplasts and cells to statistically analyse their movement. The microbeam and time-lapse video-recording systems are often used with this method. For example, cells are partly irradiated by a microbeam of weak blue light, chloroplast movements are then recorded by time-lapse imaging, and their positions are tracked using the obtained images. This method has been used to estimate the transfer speed of unknown signals by measuring the time at which chloroplast movement began after blue light irradiation in *A. capillus-veneris* and *A. thaliana* [13, 14]. The signal transfer speed for these species was determined to be 1 µm/min and 0.7 µm/min, respectively, at 25 °C. In previous studies, the start time of chloroplast movement during the accumulation response was determined by visual inspection [13–15]. As chloroplasts constantly move by a random walk, this method is somewhat analyst-dependent. Therefore, a method of objective estimation is required, which is independent of the analyst.

The statistical formulation of dynamic systems can be given by state-space models which represent the observations and underlying true state of the system. State-space modelling is often applied to time-series data to understand trends and oscillations of the system and the influence of external drivers as well as to predict the future dynamics [16, 17]. State-space modelling incorporates the observation error and system noise which are often assumed to follow Gaussian distributions. In certain state-space models, linear Gaussian models, parameters and hidden states can be sequentially estimated using the Kalman filter and maximum likelihood inference [18]. For more general state-space models which include both nonlinear and non-Gaussian configurations, Bayesian inference of the parameters is effective [19] and provides flexible modelling although at a high calculation cost.

In this study, we developed the cellssm package of R (https://github.com/hnishio/cellssm.git), which is a user-friendly tool for state-space modelling of the directional movements of cells and organelles. Our method showed a high accuracy in estimating the start time of chloroplast movement in *M. polymorpha* over a short period. We found that chloroplast movement accelerated during transport to the light-irradiated area, the majority of the time required for chloroplasts to begin movement related to signal transfer time, and signal transfer speed was uneven within a cell. We also showed that the cellssm package could be applied to the accumulation response of a nucleus and the computer-simulated *Paramecium* escape response. We demonstrated that state-space modelling is a powerful method for evaluating directional movements at cellular and subcellular levels.

## Results

### Estimation of the start time of chloroplast movement during the accumulation response

**Fig. 1:**
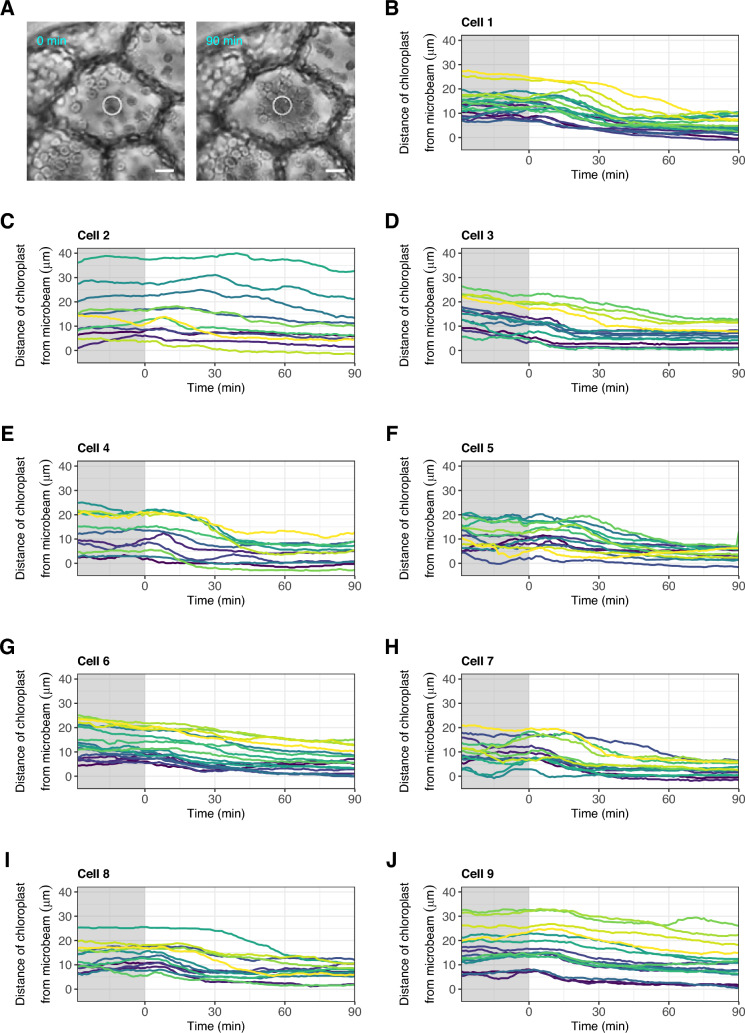
Chloroplast accumulation response in *M. polymorpha*. **A** Representative photographs at 0 min (left) and 90 min (right) after continuous irradiation with a blue microbeam (10 μm in diameter, 1 W/m^2^) for 90 min. White circles indicate irradiated areas. Scale bars represent 10 μm. **B**–**J** Temporal changes in the distance of chloroplasts from the blue microbeam in cell 1–cell 9. In each graph, coloured lines signify different chloroplasts in each cell. The shaded and light regions are the periods under the observation light and under blue microbeam irradiation, respectively

Using a temperature-regulated microscope with a microbeam system [20, 21], we obtained time-lapse images of chloroplasts in nine gemmaling cells, each derived from different individuals of *M. polymorpha*. The samples were kept at 22 °C before and after irradiation with a weak blue microbeam (1 W/m^2^) for 90 min (Fig. 1). These cells were placed under the observation light (whole cell irradiation with red light) throughout the time course. We tracked the position of the chloroplast centre on the time-lapse images and measured the distance between the chloroplast position and the edge of microbeam to monitor the chloroplast movement. The chloroplasts moved towards the microbeam-irradiated area (accumulation response) in these cells (Fig. 1A). The distance of the chloroplasts from the microbeam fluctuated before irradiation, indicating that their positions changed following a random walk under the observation light (Fig. 1B–J), which is consistent with previous reports [12]. After irradiation began, the distance decreased after certain time lags, and the time when the decline started varied among chloroplasts (Fig. 1B–J), which is also consistent with previous studies [13, 14, 22].

**Fig. 2:**
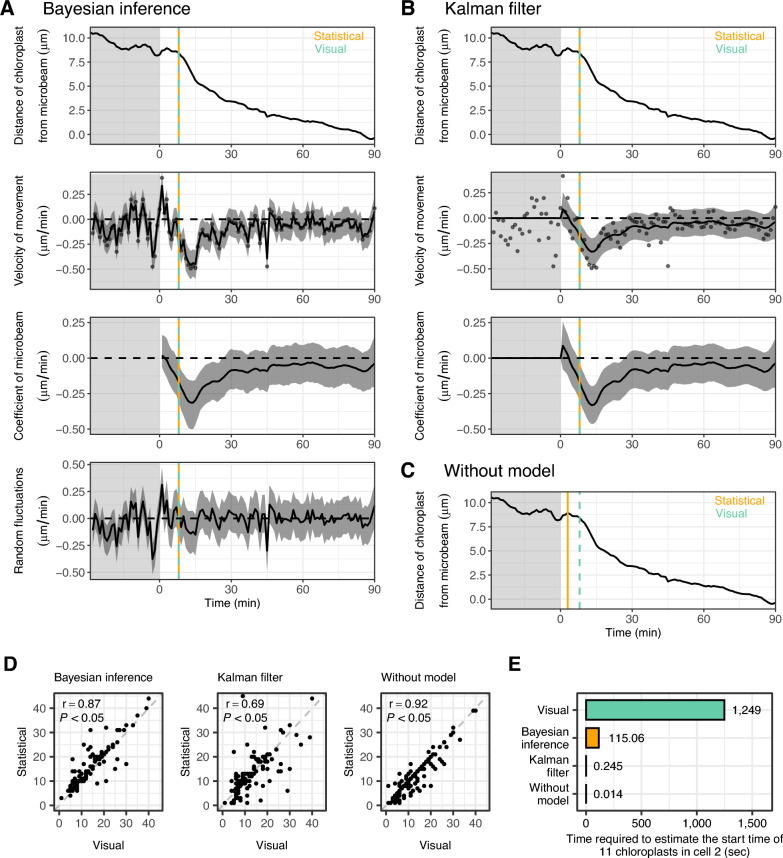
Estimation of the start time of chloroplast movement using three approaches. **A** Bayesian inference of the state-space model for a chloroplast in cell 1. The observed distance of the chloroplast from the blue microbeam (first panel), the observed and inferred velocity of movement (second panel), the inferred coefficient of the blue microbeam (third panel), and the inferred random fluctuations of the velocity (last panel) are shown. **B** Estimation of the state-space model for a chloroplast in cell 1 using the Kalman filter. The observed distance of the chloroplast from the blue microbeam (first panel), the observed and inferred velocity of movement (second panel), and the inferred coefficient of the blue microbeam (third panel) are shown. **C** Estimation of the start time for a chloroplast in cell 1 without the state-space model. The observed distance of the chloroplast from the blue microbeam is shown. **D** Comparison of the start time of chloroplast movement between the visual estimation and the three methods. Each dot represents the data point of each chloroplast. **E** Comparison of the total time required to estimate the start time of the movement of 11 chloroplasts in cell 2 between the visual estimation and the three methods. In the panels including inferred values, dots, solid lines, and shaded regions are the observed values, medians, and 99% credible intervals of the Bayesian inference, respectively. In **A**–**C**, orange solid lines and green dashed lines represent the start time estimated by each method and the visual observation, respectively. The shaded and light regions are the periods under observation light and under blue microbeam irradiation, respectively

To statistically estimate when chloroplast movement began in the accumulation response, we used state-space modelling combined with Bayesian inference. The locations of chloroplasts were assumed to be determined by the effect of the blue microbeam and their random walk throughout the time course. Because changes in the location of chloroplasts result from changes in their velocity, we attempted to explain the velocity of chloroplast movement using the influence of the microbeam and random fluctuations (Fig. 2A and Additional file 1: Fig. S1, S2, S3A). The coefficient of microbeam was mainly negative throughout the time course with troughs after certain time periods (Fig. 2A and Additional file 1: Fig. S1, S2, S3A). We defined the start time of chloroplast movement as the time when the 99% upper bounds of the time-varying coefficient of microbeam first became negative (Fig. 2A and Additional file 1: Fig. S1, S2, S3A). If no negative values occurred at any time point, the threshold was sequentially lowered to the 95% and 90% upper bounds.

Bayesian inference of state-space models requires a high computational cost for sampling from and/or estimating the posterior distributions of parameters. In this study, the Markov Chain Monte Carlo (MCMC) approach [19] was used to sample from the posterior distributions, while approximate computation of posteriors can be implemented by, for example, variational Bayes [23, 24] and Laplace approximation [25, 26]. To reduce the computational cost of Bayesian approaches and ensure user convenience, we used a state-space model without random fluctuations of the movement velocity and estimated the parameters using the Kalman filter. The movement velocity was explained only by the influence of the microbeam, while the start time of chloroplast movement was defined in the same manner as that of the Bayesian approach (Fig. 2B and Additional file 1: Fig. S1, S2, S3B).

We also estimated the start time of chloroplast movement without using the state-space models (without-model approach). In this approach, we focused on the distances of chloroplasts from the microbeam and defined the start time of chloroplast movement as the time when the three criteria described in the Methods section were first met (Fig. 2C and Additional file 1: Fig. S1, S2, S3C).

The Bayesian and without-model approaches agreed closely with the start time of chloroplast movement determined by visual estimations (Pearson’s correlation coefficient > 0.87, with visual estimations; Fig. 2D). The Kalman filter approach showed weaker correlation (Pearson’s correlation coefficient = 0.69). The time required to estimate the start time was considerably reduced using these methods (Fig. 2E). The start time of chloroplast movement determined by the without-model approach was used for the analyses in Figs. 4–7, because it showed a better correlation with visual estimations than other approaches (Fig. 2D).

### Acceleration of chloroplast movement

**Fig. 3:**
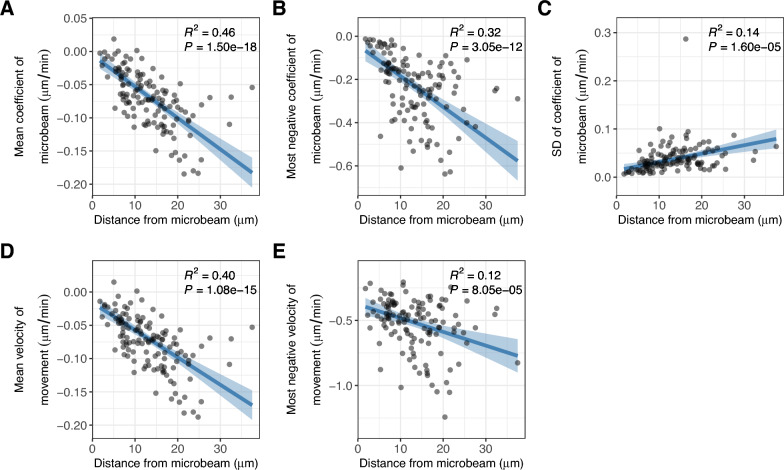
Variability in the velocity of chloroplast movement along the distance from a blue microbeam. **A**–**E** Repeated median regression of the mean coefficient of microbeam during the time course (**A**), the most negative coefficient of microbeam (**B**), the standard deviation of coefficient of microbeam (**C**), the mean velocity of movement (**D**), and the most negative velocity of movement (**E**), against the distance of the chloroplast from the blue microbeam at the start of irradiation. In **A**–**E**, dots, solid lines, and shaded regions are the observed values, regression lines, and 95% confidence intervals, respectively

To test if the velocity of chloroplast movement during transport depends on the distance from the blue microbeam at the start of irradiation, we performed repeated median regression [27]. We found that the mean and the most negative (minimum) coefficients of microbeam were explained by the distance from the microbeam (negative regression coefficients, *P* < 0.001, Fig. 3A, B). The standard deviation of the coefficient of microbeam was also explained by the distance from the microbeam (positive regression coefficients, *P* < 0.001, Fig. 3C). These results suggest that the influence of the microbeam on chloroplast velocity increased in direct proportion to the distance from the microbeam. In addition, the mean and the most negative (minimum) velocity of movement were explained by the distance from the microbeam (negative regression coefficients, *P* < 0.001, Fig. 3D, E). These results suggest that the velocity of movement increased and thus the movement accelerated during transport to the microbeam-irradiated area.

### Signal transfer speed estimated by linear regression

**Fig. 4:**
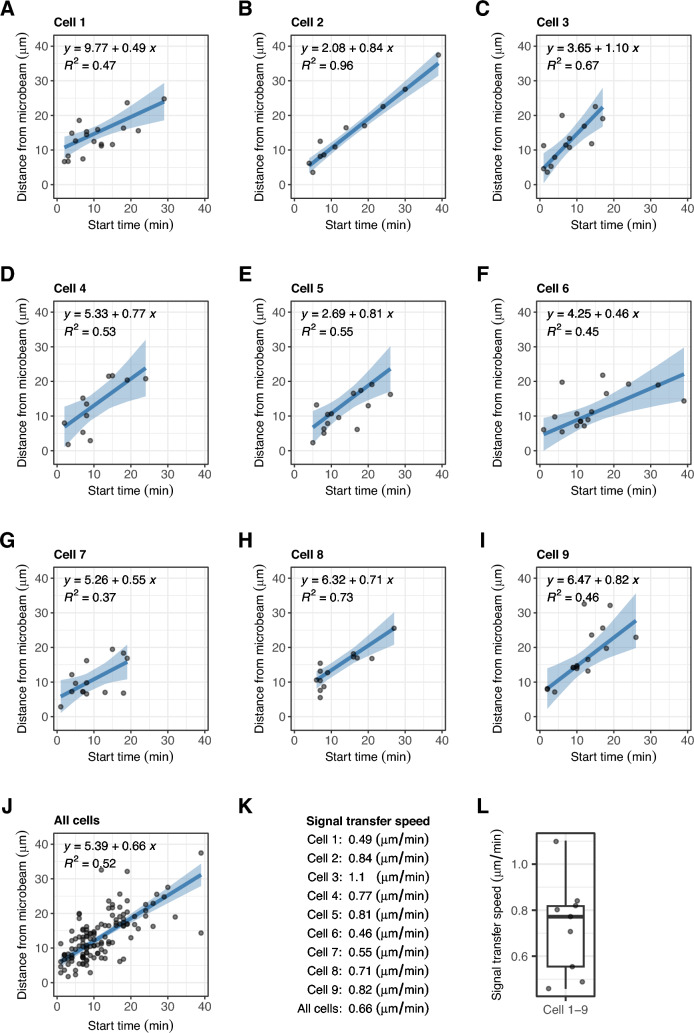
Estimation of the signal transfer speed by repeated median regression. **A**–**J** Repeated median regression of the distance of chloroplasts from the blue microbeam, against the start time of chloroplast movement estimated by the without-model approach, in cell 1–cell 9 and all cells. The signal transfer speed was defined as the slope of the regression lines. Dots, solid lines, and shaded regions are the observed values, regression lines, and 95% confidence intervals, respectively. **K** List of the estimated signal transfer speed. **L** Boxplot of the estimated signal transfer speed in cell 1–cell 9. A box represents quartiles, a centre line the median, and whiskers extend to the highest and lowest values within 1.5 × interquartile range

We performed repeated median regression [27] to estimate the speed of signal transfer from the microbeam-irradiated area to chloroplasts in each cell (Fig. 4). When using the distances of chloroplasts from the microbeam at the start of irradiation as a response variable and the start time of chloroplast movement as an explanatory variable, the slope of the regression line represents the signal transfer speed in each cell (Fig. 4). The estimated signal transfer speed varied among the nine cells ranging from 0.46 to 1.1 μm/min (Fig. 4A–K). The median signal transfer speed was 0.77 μm/min in *M. polymorpha* gemmaling cells (Fig. 4L).

**Fig. 5:**
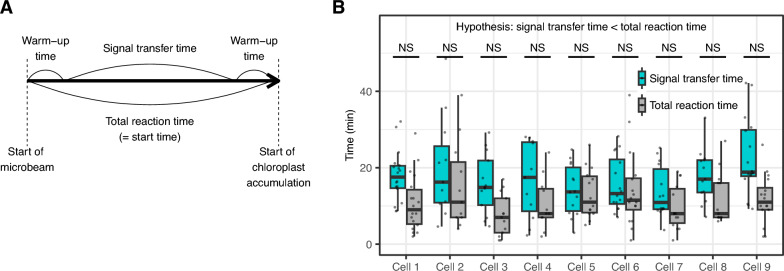
Comparison between the signal transfer time and the total reaction time. **A** The relationship between the total reaction time (= start time), the signal transfer time, and the two warm-up times. **B** Comparison between the signal transfer time and the total reaction time for the nine cells. Wilcoxon rank sum test (one-sided; signal transfer time < total reaction time) was performed. *NS* not significant

If warm-up times occurred, that is, time periods before and after signal transfer from the activated phototropin to chloroplasts, the total reaction time (start time) was assumed to be the sum of the signal transfer time and the two warm-up times (Fig. 5A). To test the presence of warm-up times, we calculated the signal transfer time in the nine cells by dividing the chloroplast distance from the microbeam by the signal transfer speed. In these cells, the signal transfer time was not less than the total reaction time (Fig. 5B). Thus, the warm-up times could be too short to be detected by the 1-min interval data.

### Signal transfer speed estimated by pairwise comparison

**Fig. 6:**
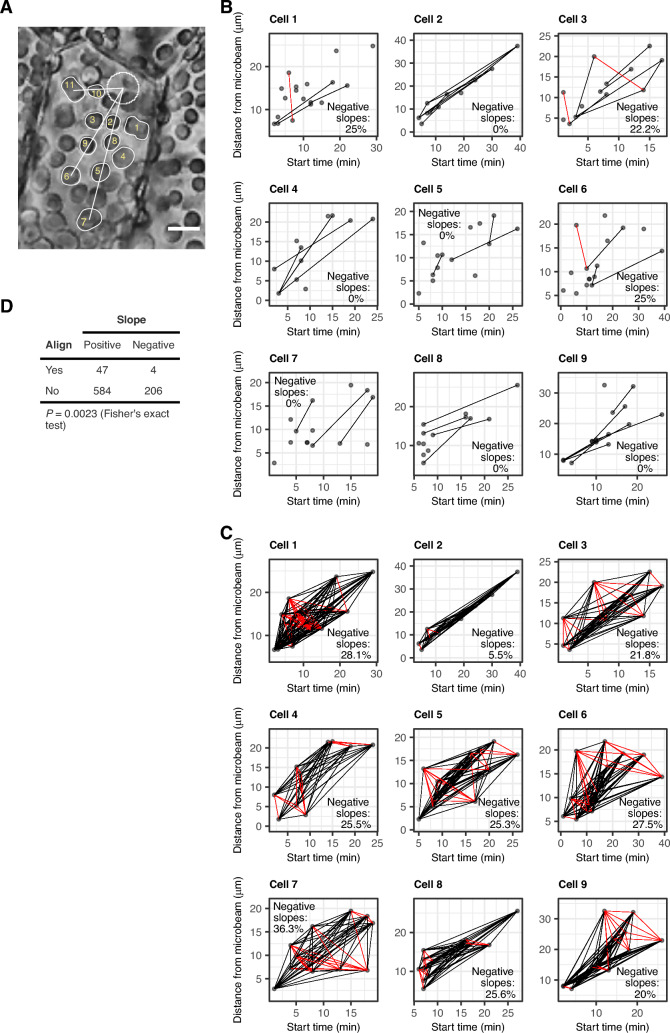
Estimation of the signal transfer speed by pair-wise comparison of chloroplasts. **A** An example of chloroplast pairs in a line (cell 2). **B** The signal transfer speed (slope) for chloroplast pairs in a line. **C** The signal transfer speed (slope) for all combinations of chloroplast pairs. In **B** and **C**, dots represent the estimated values by the without-model approach. Black and red lines represent the chloroplast pairs with positive and negative slopes, respectively. The percentage of negative slopes is shown in the plots. **D** Contingency table between alignment and sign of slope where the number of chloroplast pairs are shown. Fisher’s exact test (two-sided) was performed

As an alternative method to estimate the signal transfer speed, we used a pairwise comparison of the chloroplasts by dividing the difference in the chloroplast distance from the microbeam by the difference in the start time of chloroplast movement (Fig. 6). When we calculated the signal transfer speed for chloroplast pairs in a line (align pairs) (Fig. 6A), the signal transfer speeds (slopes in Fig. 6B) were positive for most pairs except for four pairs in three cells (Cells 1, 3, and 6) (Fig. 6B). In contrast, when we calculated the signal transfer speed for all combinations of chloroplast pairs (non-align pairs), most signal transfer speeds (slopes in Fig. 6C) were positive but some were negative in all cells (Fig. 6C). The number of negative slopes in the non-align pairs were significantly larger than that in the align pairs (*P* = 0.0023, Fisher's exact test, Fig. 6D). Thus, in some chloroplast pairs, the more distant chloroplast responded to the microbeam earlier than the closer partner (Fig. 6C). These results suggest that the signal was transferred linearly in one direction, but the transfer efficiency was not uniform among different directions.

### Common dynamics of chloroplasts in each cell

**Fig. 7:**
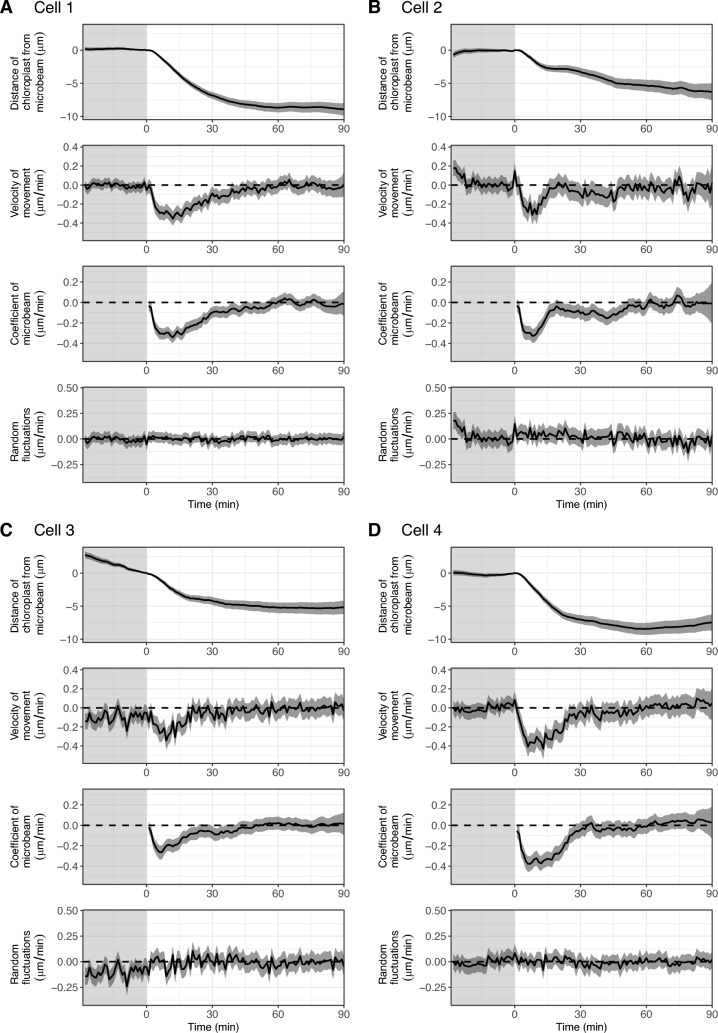

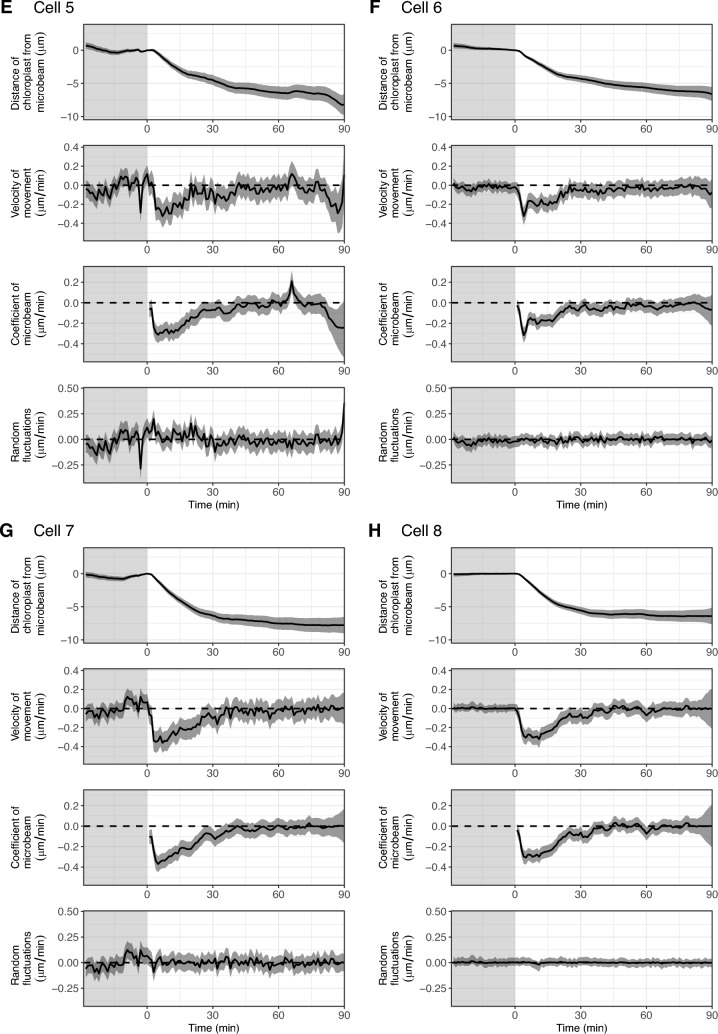

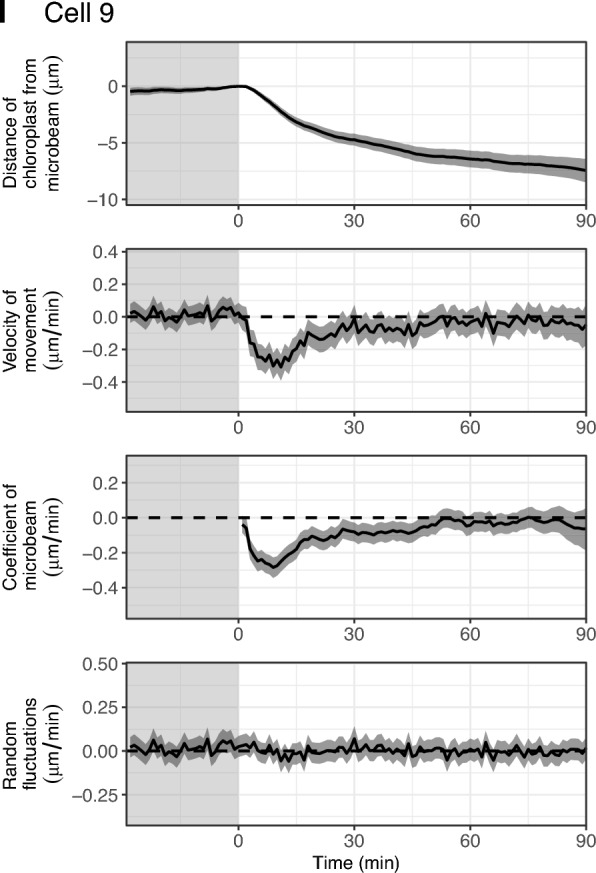
Estimation of the common dynamics of chloroplast movements using the state-space model. **A**–**I** Bayesian inference of the state-space model assuming the common dynamics between chloroplasts for cell 1–cell 9. The estimated values of the distance of the chloroplast from the blue microbeam (first panel), the velocity of movement (second panel), the coefficient of the blue microbeam (third panel), and the random fluctuations of the velocity (last panel) are shown. Solid lines and shaded regions are the medians and 95% credible intervals of the Bayesian inference, respectively. The shaded and light regions are the periods under observation light and under blue microbeam irradiation, respectively

To estimate the representative dynamics of chloroplasts in each cell, we developed a ‘common model’ using state-space modelling. For each cell, we assumed an imaginary chloroplast which had a distance from the microbeam of zero, and the dynamics (position and velocity) of the chloroplast is referred to as the ‘common dynamics’. We then assumed that the observed dynamics of all chloroplasts were derived from the ‘common dynamics’ with modifications of (1) the time-lag of the start time of chloroplast movement depending on the distance from the microbeam, and (2) system noise and observation error. With these assumptions, we estimated the ‘common dynamics’ for each cell (Fig. 7). We observed the similar dynamics among cells: velocity of movement reached the minimum from − 0.2 to − 0.4 μm/min within 15 min after irradiation began (Fig. 7). We also detected the slightly different characteristics between the cells: for example, the velocity of movement was negative for a long period in cell 2, indicating that chloroplasts gradually approached the microbeam in this cell (Fig. 7B), and the most negative (minimum) velocity of movement was lowest in cell 4, indicating that chloroplasts approached the microbeam most rapidly in this cell (Fig. 7D).

### Application of the developed method to another organelle and a microbe

To test if the developed cellssm package could be applied to different dataset types, we first applied the method to the accumulation response of a nucleus to light [28, 29]. The time-lapse images of a gemmaling cell of *M. polymorpha* were obtained to track the position of the nucleus in the same experimental condition as chloroplast accumulation response using a blue microbeam. We could capture the dynamics and determine the start time of nucleus movement, using the Bayesian, Kalman filter, and without-model approaches (Additional file 1: Fig. S4). Next, we created a simulated data by computer to imitate the escape response of a microbe *Paramecium* to laser heating [30], where we assumed the laser heating was applied for 70 min during the 200 min observation period. We could capture the dynamics and determine the start time of the escape response using the three approaches (Additional file 1: Fig. S5). Thus, the developed cellssm package can be generally applied to directional movements of organelles and microbes in both accumulation and escape responses.

## Discussion

In previous studies, much of the data on chloroplast position has been manually analysed using visual judgement to analyse the start time of chloroplast movement in the accumulation response [13–15]. This manual analysis created differences depending on analyst, and required a considerable investment in time. The cellssm package of R provides statistical methods based on state-space modelling to determine the start time of organelle movement, allowing for objective estimations. Notably, our method showed a high accuracy to judge the start time of chloroplast movement (high correlation with visual estimations; Fig. 2A–D). In addition, the analysis time was reduced by over 90% from the time required for visual estimation (Fig. 2E). Thus, the cellssm package is an accurate and rapid tool to analyse organelle movements.

Using this tool, we found that the most negative velocity of chloroplast movements during the test period was explained by the distance from the microbeam. This result is consistent with a study on *A. capillus-veneris* which stated that the maximum speed of chloroplasts located farther from a microbeam is greater than that of those nearer to the beam [13]. These results suggest that the velocity of chloroplast movement induced by a microbeam is not constant but increases during the transport along cp-actin filaments. Similar to a car driving on a highway, travelling longer distances allows for higher speeds than travelling shorter distances.

The speed of the unknown signal travelling from phototropin at the light-irradiated area to the chloroplasts in *M. polymorpha* gemmaling cells was 0.77 μm/min at 22 °C (Fig. 4L). This speed is similar to previously reported signal transfer speeds in *A. capillus-veneris* (1 μm/min) and *A. thaliana* (0.7 μm/min) at 25 °C [13, 14]. Although the speed depends on temperature [14], the speed showed little variation among the tested plant species.

Most of the time required for the accumulation response is used for the signal transfer (Fig. 5). The total time for the response consists of three periods: phototropin-mediated warm-up at the light-irradiated area (Period 1), transfer time of the unknown signal (Period 2), and cp-actin-mediated warm-up on the chloroplast before movement (Period 3). Based on our results with time-lapse imaging at 1 min intervals, warm-up times at the light-irradiated area (Period 1) and on the chloroplast (Period 3) appeared to be less than 1 min. Consistent with our data, a previous study reported that phototropin from *M. polymorpha* is activated (autophosphorylated) within 1 min after blue light irradiation [31]. Note that cp-actin for the accumulation response was observed only at 5 min intervals in the study with *A. thaliana* [9]. To estimate the warm-up times, time-lapse analyses with intervals less than 1 min are required.

The signal for the accumulation response was not transferred uniformly among different directions (Fig. 6). In a previous study with *A. capillus-veneris* using red microbeams, the signal transfer speed was predicted to be generally uniform regardless of the presence of obstacles such as other organelles [14]. Our results indicated that the signal was transferred linearly in one direction, and the transfer efficiency was uneven among different directions in *M. polymorpha*. The signal transfer may be influenced by the condition of each passage in the cell, for example, the presence of other organelles, cytoskeletal polymers, proteins, and other cellular components may inhibit or promote the signal transfer.

One of the functions in the cellssm package is a model to estimate the common dynamics of organelles in each cell, which clarifies different characteristics between cells. This model would be useful for purposes such as visualising the true differences in the dynamics of cells or organelles between mutant and wild type cells behind messy data sets represented by different positions of cells or organelles and various noise.

## Conclusions

Our study showed that state-space modelling is useful to statistically analyse chloroplast movements in the accumulation response. As we have demonstrated, the cellssm package can be applied to other directional movements (both accumulation and avoidance) at cellular and subcellular levels, such as chemo-, photo-, and thermotaxis of bacteria, and mitochondria and nucleus transport in eukaryotic cells. Thus, our tool would be useful to estimate the true transition of states behind time-series data with random fluctuations in various biological phenomena.

## Methods

### Plant materials and growing conditions

*Marchantia polymorpha* (the male accession Takaragaike-1: Tak-1) was asexually maintained on ½ B5 medium with 1% (w/v) agar (BOP, SSK Sales Co., Ltd., Shizuoka, Japan) under continuous white fluorescent light of approximately 70 µmol photons m^–2^ s^–1^ (FL40SW, NEC Corporation, Tokyo, Japan) in a culture room at 22 °C [32]. For the analysis of the accumulation response of chloroplasts, 3-day-old gemmalings (thalli grown from gemmae) were used. Gemmae were precultured for 2 days at 22 °C under continuous red LED light of 25 µmol photons m^–2^ s^–1^ (660 nm, ISL-150 × 150-H4RB, CCS Inc., Kyoto, Japan) in an incubator (IJ100 and IJ101, Yamato Scientific Co., Ltd., Tokyo, Japan). The 2-day-old gemmalings were then incubated for 1 day at 22 °C in the dark to induce the dark positioning of chloroplasts, during which time the chloroplasts move towards the anticlinal wall, reducing the number of chloroplasts at the periclinal wall [31]. The light intensity was measured with a light meter (LI-250A, LI-COR Biosciences, Lincoln, NE, USA).

### Measurement of chloroplast locations

To induce the accumulation response, we used a temperature-regulated microscope with a blue microbeam [20, 21] and a time-lapse video-recording system (Moticam2000, Shimadzu RIKA Corporation, Tokyo, Japan). The gemmaling was mounted onto glass slides in hydrogel to prevent movement under the microscope [33, 34]. The gemmaling was maintained at 22 °C under red light (300 µmol photons m^–2^ s^–1^) as observation light, and cell images were acquired for 120 min at 1 min intervals. The red-light intensity was measured with a light meter (LI-250A, LI-COR Biosciences). After recording the images under the red-light for 30 min, a cell was irradiated with 1 W m^–2^ of blue microbeam (diameter: 10 µm) to induce the accumulation response of chloroplasts, and images were further acquired for 90 min. The collimated blue light was obtained from a blue coloured LED fibre at 450.8 nm (FOLS-01, Pi Photonics, Inc., Hamamatsu, Japan) through a 20 × objective lens (NA0.4), and the blue light intensity was measured using a power meter 1918-R (Newport Corporation, CA, USA) with a silicon detector 918D-SL-OD1 (detector active area: 1 cm^2^) (Newport Corporation). The position of chloroplast centre was tracked on the time-lapse images, and the distance of a chloroplast from the blue microbeam was defined to be the length of the line connecting the centre of the chloroplast to the edge of the microbeam, and was measured using ImageJ/Fiji [35]. A total of 127 chloroplasts were analysed in nine gemmaling cells, each derived from different individuals of *M. polymorpha*.

### Visual estimation of the start time of chloroplast movement during the accumulation response

To visually estimate the start time of chloroplast movement after microbeam irradiation, we opened the time-lapse images as stack images using ImageJ and tracked the trajectories of chloroplasts during the accumulation response. We visually determined the start time as the time point at which the chloroplasts began moving toward the microbeam-irradiated area.

### Computing equipment

All computations were performed on MacBook Air (Apple M1 chip, 2020) with 16 GB RAM, using R (version 4.1.1).

### State-space model to estimate the dynamics of each chloroplast (individual model)

We assumed that the distances of chloroplasts from the blue microbeam was divided into their random walk and the effect of the microbeam. The microbeam was expected to affect the velocity of chloroplast movement, thereby changing their locations. The state-space model to analyse the time-varying effect of a microbeam on the velocity of movement (Fig. 2) is defined by the following equations:

1 $$w\left[t\right] \sim Normal(0, {\sigma }_{w}^{2})$$

2 $$beam\left[ t \right]~\sim ~\left\{ \begin{gathered} 0\,\,\,\,\,\,\,\,\,\,\,\,\,\,\left( {1 \le t \le 29} \right) \hfill \\ 1\,\,\,\,\,\,\,\,\,\,\,\,\,\,\,\left( {30 \le t \le 119} \right) \hfill \\ \end{gathered} \right.$$

3 $${\beta }_{beam}\left[t\right] \sim \left\{\begin{array}{ll}Normal\left(0, {\sigma }_{\beta beam}^{2}\right) & (t=30)\\ Normal\left({\beta }_{beam}\left[t-1\right], {\sigma }_{\beta beam}^{2}\right) & (31\le t\le 119)\end{array}\right.$$

4 $$\alpha \left[ t \right] = \left\{ \begin{array} {l} w\left[ t \right]\qquad \qquad \qquad \qquad \quad \qquad \left( {1 \le t \le 29} \right) \hfill \\ w\left[ t \right] + \beta _{{beam}} \left[ t \right]\, \cdot \,beam\left[ t \right]\qquad \left( {30 \le t \le 119} \right) \\ \end{array} \right.$$

5 $$y\left[t\right] \sim Normal(\alpha \left[t\right], {\sigma }_{y}^{2})$$

where $w[t]$ is white noise at time $t$; $beam\left[t\right]$ is the absence and presence of a blue microbeam at time $t$ represented by 0 and 1, respectively; ${\beta }_{beam}\left[t\right]$ is the time-varying regression coefficient of microbeam at time $t$, which is not defined for $1\le t\le 29$; $\alpha \left[t\right]$ is the true state of velocity of movement at time $t$; $y\left[t\right]$ is the observed velocity of movement at time $t$; and ${\sigma }^{2}$ is the variance. The values of $t=(1, 2, \cdots , 119)$ are the time points at 1 min intervals. Blue microbeam irradiation began at $t=30$ and continued to $t=119$.

For the Bayesian inference of the parameters, the statistical models were written in the Stan language and the programs were compiled using CmdStan (version 2.29.2). To operate CmdStan, the cmdstanr package (version 0.5.2) of R was used. After 1000 warm up steps, 1000 MCMC samples were obtained for each of four parallel chains to obtain 4,000 MCMC samples in total. For the estimation of the parameters by the Kalman filter [18], we used the KFAS package (version 1.4.6) [36] of R without the assumption of the white noise ($w$).

The start time of chloroplast movement was estimated as the time when the 99% upper bounds of the time-varying coefficient of microbeam first became negative. If no negative results occurred at any time point, the threshold was sequentially lowered to the 95% and 90% upper bounds. If the start time was still not able to be determined, it was set to infinity for the chloroplast and not used for the downstream analyses. The estimated start time was compared with that of the visual observation of images.

### Without-model estimation of the start time of chloroplast movement

In the estimation without a statistical model, the start time of chloroplast movement was determined to be the time when the following three criteria were first met. First, the chloroplast was approaching the microbeam, that is, the distance from the microbeam decreased for three consecutive time points. Second, the chloroplast was approaching the microbeam in the long-term, that is, the moving average of the change in the distance for nine time points (1/10 of the microbeam irradiation time) was negative. Third, the influence of the microbeam was strong, that is, the decrease of the distance in 13 time points ahead was 1.5 times larger than the mean decrease. These parameters were optimised by grid searches to obtain the lowest RMSE between the start times of chloroplast movement determined by this method and visual estimation. The calculation was performed using R.

### Repeated median regression of the velocity of movement against the distance from microbeam

For each chloroplast, the mean and the most negative coefficient of microbeam were calculated as the mean and the most negative values of the medians (90 data points) of the Bayesian inference of the ‘individual model’ during the time course. The standard deviation of the coefficient of microbeam was calculated as the median of the Bayesian inference of the same model. The mean and the most negative velocity of movement were calculated in the same manner. The repeated median regression was performed using the RobustLinearReg package (version 1.2.0) of R.

### Signal transfer speed estimated by repeated median regression

Using the start time of chloroplast movement estimated by the without-model approach as an explanatory variable and the distance from the microbeam at the start of irradiation as a response variable, we performed repeated median regression using the RobustLinearReg package (version 1.2.0) of R. The coefficient of regression, that is, the slope of the regression line, was used as an approximation of the signal transfer speed. We calculated the coefficient of determination (*R*^*2*^ value) as an estimate of the goodness-of-fit of the regression lines to the observed data. The signal transfer time was calculated by dividing the distance from the microbeam at the start of irradiation by the signal transfer speed. We assumed that the total reaction time (start time) was composed of the signal transfer time and the two warm-up times. The difference between the signal transfer time and the total reaction time was tested by the Wilcoxon signed rank test (one-sided; signal transfer time < total reaction time) using the wilcox.exact function of the exactRankTests package (version 0.8.35) of R with the ‘alternative’ parameter set as ‘less’.

### Signal transfer speed estimated by pairwise comparison

The differences in the start time of chloroplast movement estimated by the without-model approach and the distance between chloroplasts were calculated for all chloroplast pairs. The speed of signal transfer between chloroplasts was estimated by dividing the distance between them by the difference in the start time. A chloroplast pair was judged to be in a line when the line connecting the centre of the blue microbeam and the centre of the most distant of the chloroplast pair includes both chloroplasts (Fig. 6A). The association between alignment and the sign of signal transfer speed was tested by the Fisher’s exact test (two-sided) using the fisher.test function of the stats package (version 4.1.1) of R.

### State-space model to estimate the common dynamics of chloroplasts in each cell (common model)

In this model, we assumed an imaginary chloroplast which had a distance from the microbeam of zero, and dynamics referred to as ‘common dynamics’ for each cell. The state-space representation of the common dynamics is similar to that of the ‘individual model’ explained in the previous section. We then assumed that the real dynamics of all chloroplasts followed the common dynamics after the start time of chloroplast movement estimated for each chloroplast by the without-model approach. The state-space representation of the ‘common model’ (Fig. 7) is defined by the following equations:

6 $$w\left[t\right] \sim Normal(0, {\sigma }_{w}^{2})$$

7 $$beam\left[t\right] \sim \left\{\begin{array}{ll}0 & (1\le t\le 29)\\ 1 & (30\le t\le 119)\end{array}\right.$$

8 $${\beta }_{beam,common}\left[t\right] \sim \left\{\begin{array}{ll}Normal\left(0, {\sigma }_{\beta beam}^{2}\right) & (t=30)\\ Normal\left({\beta }_{beam,common}\left[t-1\right], {\sigma }_{\beta beam}^{2}\right) & (31\le t\le 119)\end{array}\right.$$

9 $${\beta }_{beam,each}\left[t,n\right] \sim \left\{\begin{array}{ll}0 & (30\le t\le (27+start\left[n\right]))\\ {\beta }_{beam,common}\left[t-start[n]+2\right] & ((28+start\left[n\right])\le t\le 119)\end{array}\right.$$

10 $$\alpha _{{common}} \left[ t \right] = \left\{ \begin{array}{l} w\left[ t \right]\qquad \qquad \qquad \qquad \qquad \qquad \quad \left( {1 \le t \le 29} \right) \\ w\left[ t \right] + \beta _{{beam,common}} \left[ t \right]\, \cdot \,beam\left[ t \right]\quad \left( {30 \le t \le 119} \right) \\ \end{array} \right.$$

11 $$\alpha _{{each}} \left[ {t,n} \right] = \left\{ \begin{gathered} w\left[ t \right]\qquad \qquad \qquad \qquad \quad \qquad \qquad \left( {1 \le t \le 29} \right) \hfill \\ w\left[ t \right] + \beta _{{beam,each}} \left[ {t,n} \right]\, \cdot \,beam\left[ t \right]\,\,\,\,\,\left( {30 \le t \le 119} \right) \\ \end{gathered} \right.$$

12 $$y\left[t,n\right] \sim Normal({\alpha }_{each}\left[t,n\right], {\sigma }_{y}^{2})$$

13 $${dist}_{common}\left[t\right]= \sum_{i=1}^{t}{\alpha }_{common}\left[i\right]-\sum_{i=1}^{29}{\alpha }_{common}\left[i\right]$$

where $w[t]$ is white noise at time $t$; $beam\left[t\right]$ is the absence and presence of the blue microbeam at time $t$ represented by 0 and 1, respectively; ${\beta }_{beam,common}\left[t\right]$ is the common time-varying regression coefficient of microbeam at time $t$, which is not defined for $1\le t\le 29$; ${\beta }_{beam,each}\left[t,n\right]$ is the time-varying regression coefficient of microbeam at time $t$ in the chloroplast $n$, which is not defined for $1\le t\le 29$; ${\alpha }_{common}\left[t\right]$ is the common velocity of movement at time $t$; ${\alpha }_{each}\left[t,n\right]$ is the estimated velocity of movement at time $t$ in the chloroplast $n$; $y\left[t,n\right]$ is the observed velocity of movement at time $t$ in the chloroplast $n$; ${dist}_{common}\left[t\right]$ is the common distance of the chloroplasts from the microbeam at time $t$; and ${\sigma }^{2}$ is the variance. The values of $t=(1, 2, \cdots , 119)$ are the time points at 1 min intervals. Blue microbeam irradiation started at $t=30$ and continued to $t=119$. The parameters were estimated by the Bayesian inference in the same manner as that of the ‘individual model’ explained in the previous section.

## Supplementary Information

**Additional file 1: Fig. S1.** Estimation of the start time of chloroplast accumulation response using three approaches for a chloroplast in cell 2. **Fig. S2.** Estimation of the start time of chloroplast accumulation response using three approaches for a chloroplast in cell 3. **Fig. S3.** Estimation of the start time of chloroplast accumulation response using three approaches for a chloroplast in cell 4. **Fig. S4.** Application of the developed method to the accumulation response of a nucleus in a cell. **Fig. S5.** Application of the developed method to a simulated data of the *Paramecium* escape response to laser heating.

## Data Availability

All datasets and computer codes generated in this study are available in the GitHub repository, https://github.com/hnishio/Nishio_PlantMethods_script3.git.
