# Supplementary material for: Statistical analysis of organelle movement using state-space models
Source: Plant Methods. 2023 Jul 5;19:67. doi: 10.1186/s13007-023-01038-6 (PMC10321007; doi:10.1186/s13007-023-01038-6)
Supplement: Supplementary file 1 — Additional file 1: Fig. S1. Estimation of the start time of chloroplast accumulation response using three approaches for a chloroplast in cell 2. Fig. S2. Estimation of the start time of chloroplast accumulation response using three approaches for a chloroplast in cell 3. Fig. S3. Estimation of the start time of chloroplast accumulation response using three approaches for a chloroplast in cell 4. Fig. S4. Application of the developed method to the accumulation response of a nucleus in a cell. Fig. S5. Application of the developed method to a simulated data of the Paramecium escape response to laser heating. [file 13007_2023_1038_MOESM1_ESM.pdf]

Additional file 1 for

Statistical analysis of organelle movement using state-space models

Haruki Nishio*, Satoyuki Hirano, Yutaka Kodama*

*Correspondence: harukin218@gmail.com and kodama@cc.utsunomiya-u.ac.jp

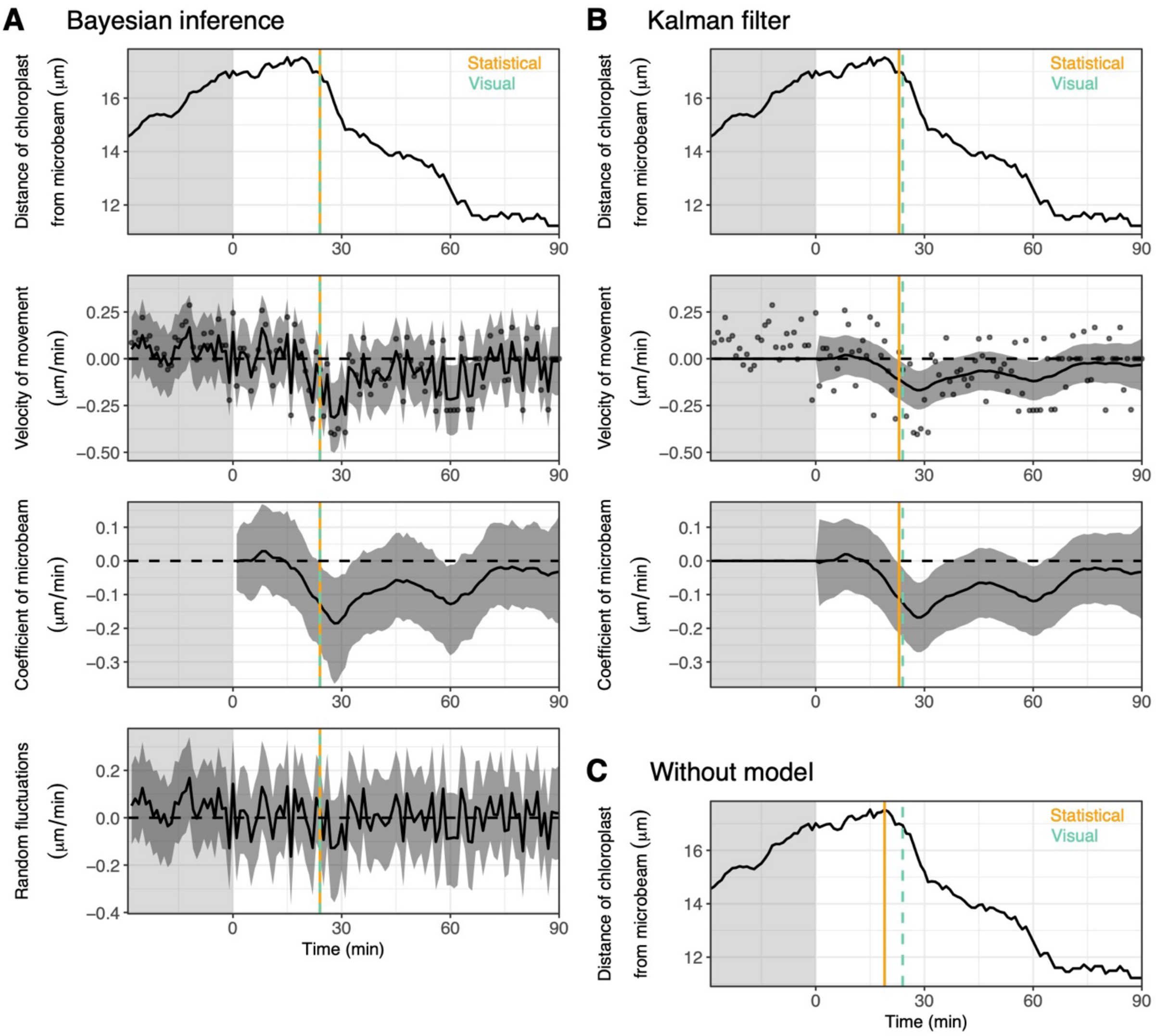

Fig. S1 Estimation of the start time of chloroplast accumulation response using three approaches for a chloroplast in cell 2. **A** Bayesian inference of the state-space model for a chloroplast in cell 2. The observed distance of the chloroplast from the blue microbeam (first panel), the observed and inferred velocity of movement (second panel), the inferred coefficient of the blue microbeam (third panel), and the inferred random fluctuations of the velocity (last panel) are shown. **B** Estimation of the state-space model for a chloroplast in cell 2 using the Kalman filter. The observed distance of the chloroplast from the blue microbeam (first panel), the observed and inferred velocity of movement (second panel), and the inferred coefficient of the blue microbeam (third panel) are shown. **C** Estimation of the start time for a chloroplast in cell 2 without the state-space model. The observed distance of the chloroplast from the blue microbeam is shown. In the panels including inferred values, dots, solid lines, and shaded regions are the observed values, medians, and 99 % confidence intervals, respectively. In **A**–**C**, orange solid lines and green dashed lines represent the start time estimated by each method and the visual observation, respectively. The shaded and light regions are the periods under observation light and under blue microbeam irradiation, respectively.

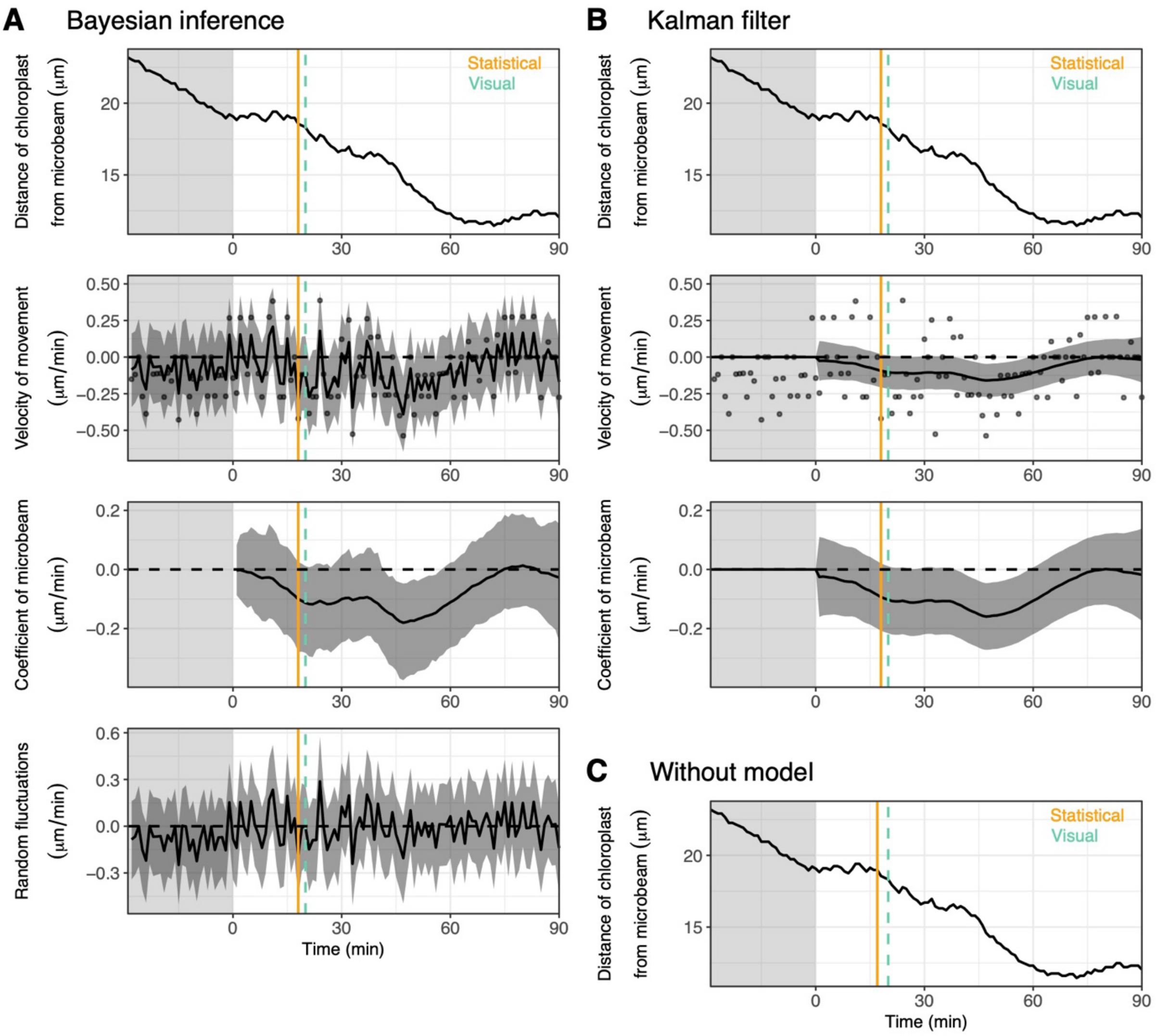

Fig. S2 Estimation of the start time of chloroplast accumulation response using three approaches for a chloroplast in cell 3. **A** Bayesian inference of the state-space model for a chloroplast in cell 3. The observed distance of the chloroplast from the blue microbeam (first panel), the observed and inferred velocity of movement (second panel), the inferred coefficient of the blue microbeam (third panel), and the inferred random fluctuations of the velocity (last panel) are shown. **B** Estimation of the state-space model for a chloroplast in cell 3 using the Kalman filter. The observed distance of the chloroplast from the blue microbeam (first panel), the observed and inferred velocity of movement (second panel), and the inferred coefficient of the blue microbeam (third panel) are shown. **C** Estimation of the start time for a chloroplast in cell 3 without the state-space model. The observed distance of the chloroplast from the blue microbeam is shown. In the panels including inferred values, dots, solid lines, and shaded regions are the observed values, medians, and 99 % confidence intervals, respectively. In **A**–**C**, orange solid lines and green dashed lines represent the start time estimated by each method and the visual observation, respectively. The shaded and light regions are the periods under observation light and under blue microbeam irradiation, respectively.

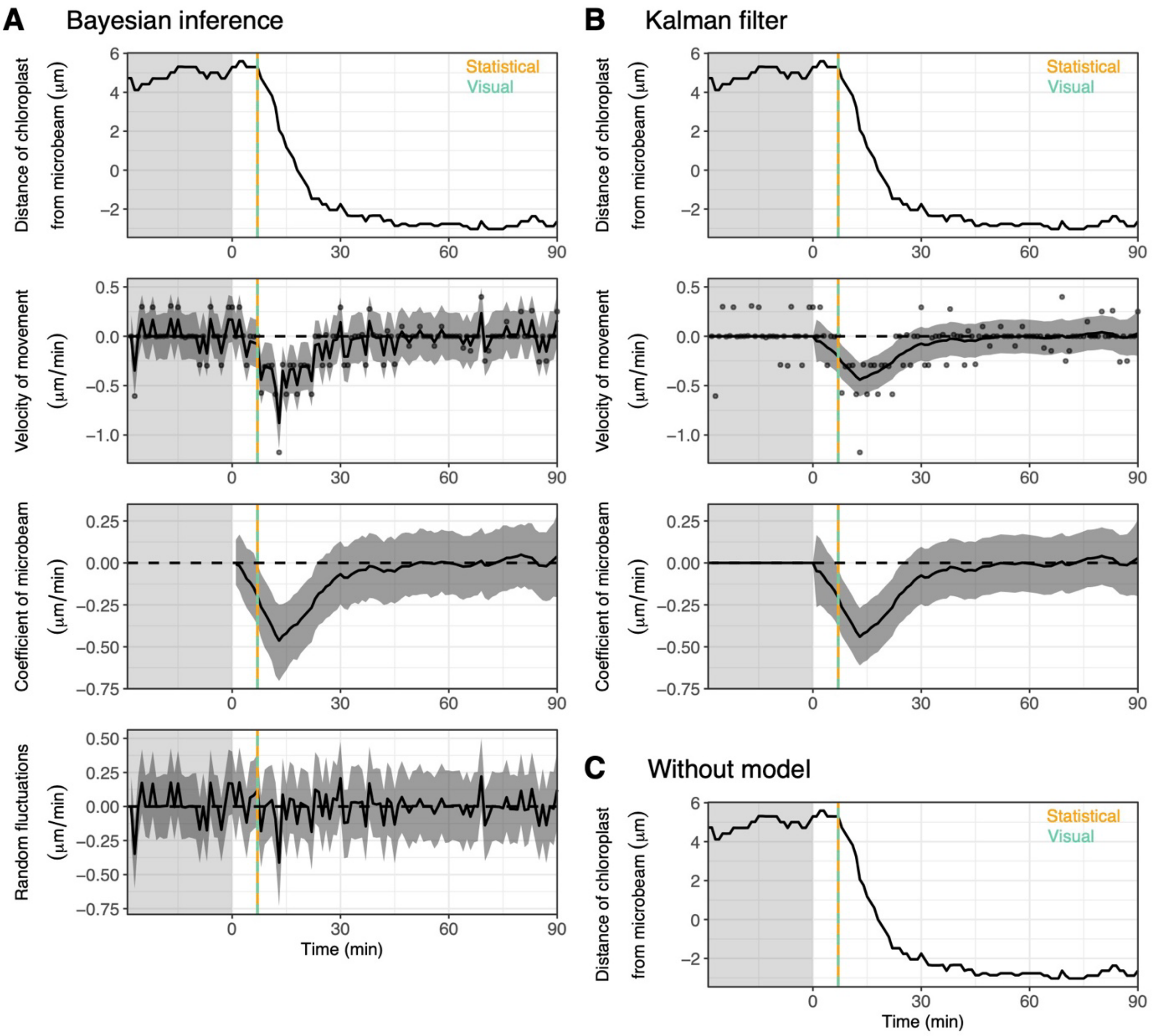

Fig. S3 Estimation of the start time of chloroplast accumulation response using three approaches for a chloroplast in cell 4. **A** Bayesian inference of the state-space model for a chloroplast in cell 4. The observed distance of the chloroplast from the blue microbeam (first panel), the observed and inferred velocity of movement (second panel), the inferred coefficient of the blue microbeam (third panel), and the inferred random fluctuations of the velocity (last panel) are shown. **B** Estimation of the state-space model for a chloroplast in cell 4 using the Kalman filter. The observed distance of the chloroplast from the blue microbeam (first panel), the observed and inferred velocity of movement (second panel), and the inferred coefficient of the blue microbeam (third panel) are shown. **C** Estimation of the start time for a chloroplast in cell 4 without the state-space model. The observed distance of the chloroplast from the blue microbeam is shown. In the panels including inferred values, dots, solid lines, and shaded regions are the observed values, medians, and 99 % confidence intervals, respectively. In **A**–**C**, orange solid lines and green dashed lines represent the start time estimated by each method and the visual observation, respectively. The shaded and light regions are the periods under observation light and under blue microbeam irradiation, respectively.

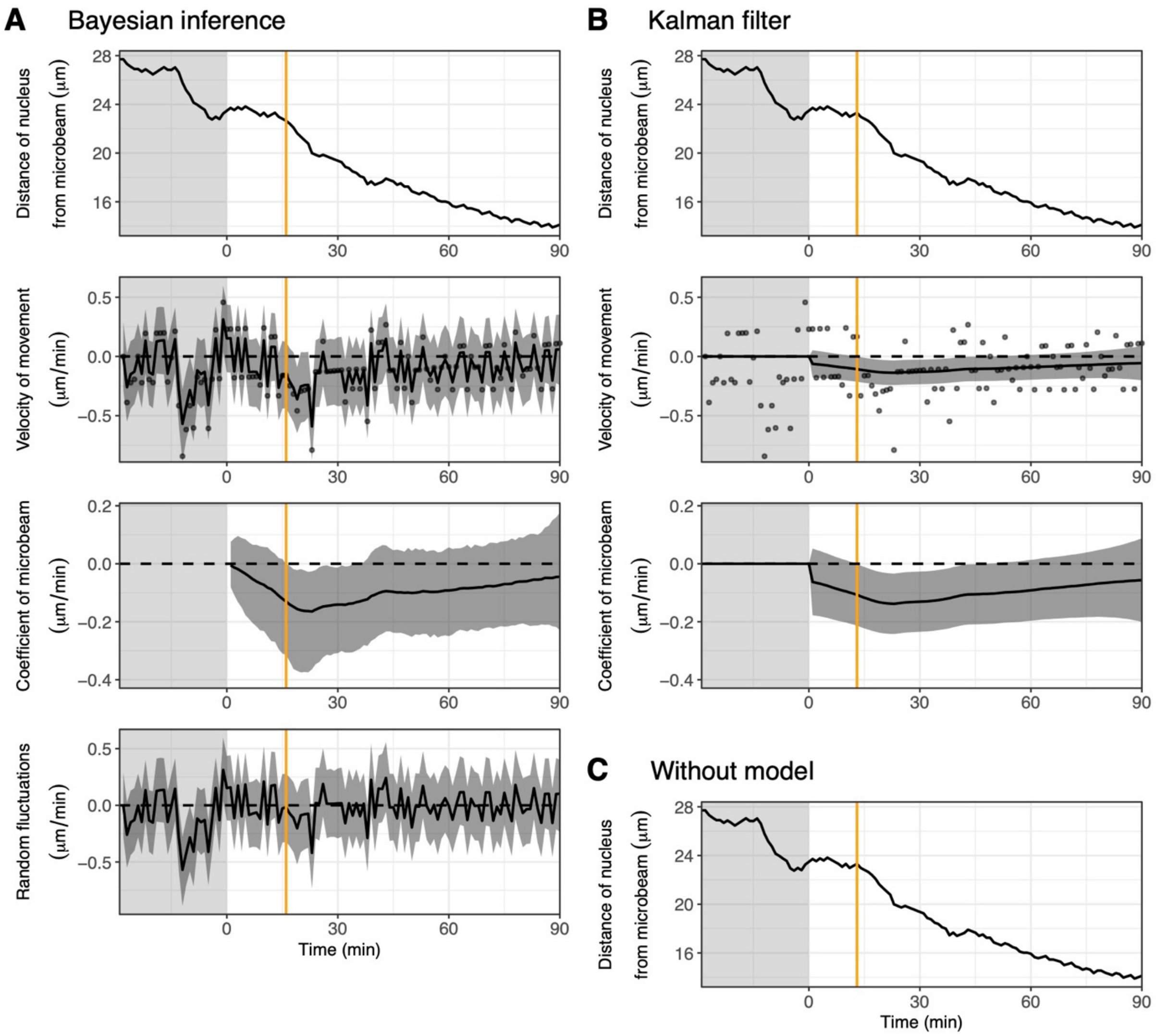

Fig. S4 Application of the developed method to the accumulation response of a nucleus in a cell. **A** Bayesian inference of the state-space model for a nucleus in a cell. The observed distance of the nucleus from the blue microbeam (first panel), the observed and inferred velocity of movement (second panel), the inferred coefficient of the blue microbeam (third panel), and the inferred random fluctuations of the velocity (last panel) are shown. **B** Estimation of the state-space model for a nucleus in a cell using the Kalman filter. The observed distance of the nucleus from the blue microbeam (first panel), the observed and inferred velocity of movement (second panel), and the inferred coefficient of the blue microbeam (third panel) are shown. **C** Estimation of the start time for a nucleus in a cell without the state-space model. The observed distance of the nucleus from the blue microbeam is shown. In the panels including inferred values, dots, solid lines, and shaded regions are the observed values, medians, and 99 % confidence intervals, respectively. In **A**–**C**, orange solid lines represent the start time estimated by each method. The shaded and light regions are the periods under observation light and under blue microbeam irradiation, respectively.

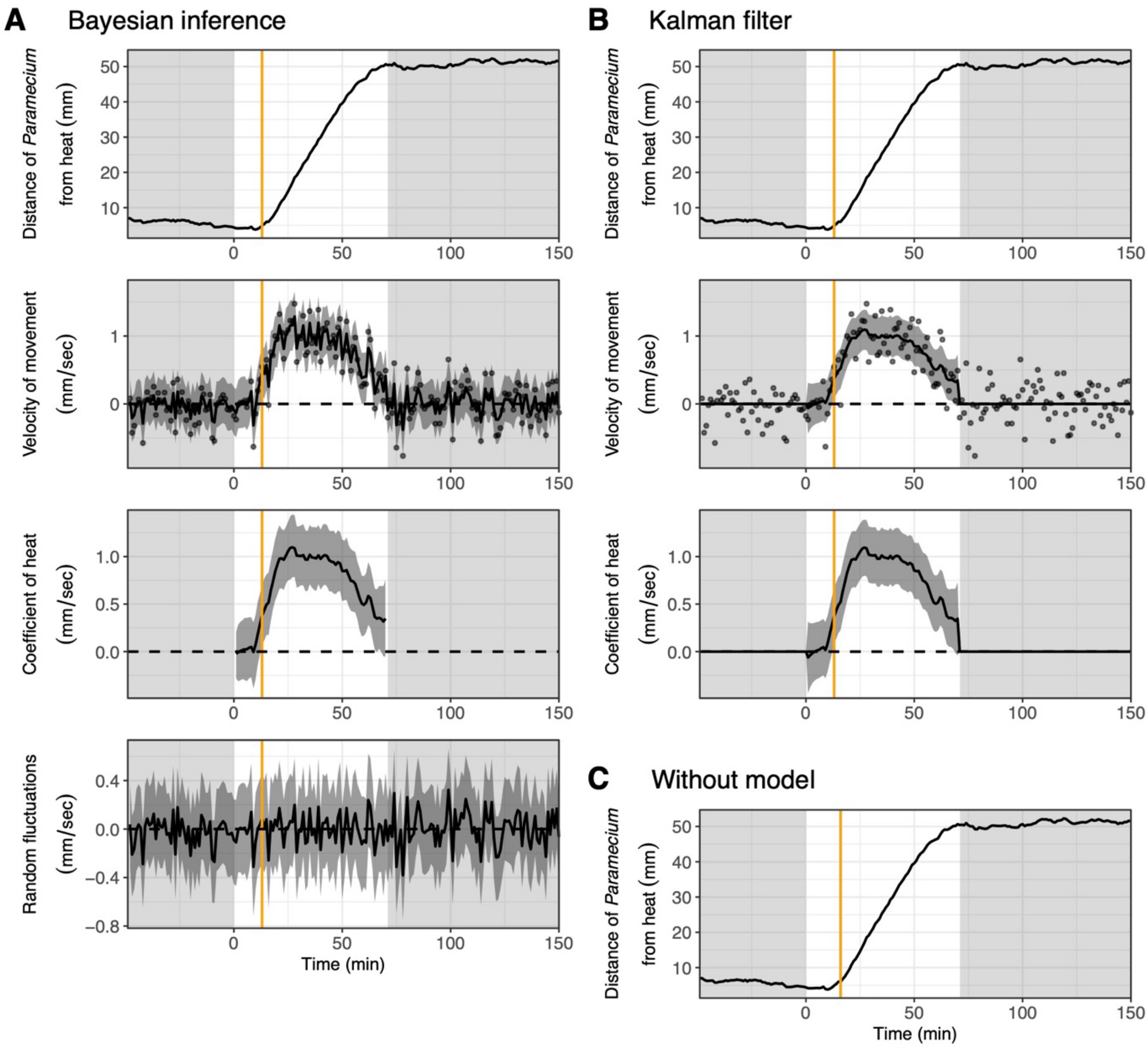

Fig. S5 Application of the developed method to a simulated data of the *Paramecium* escape response to laser heating. **A** Bayesian inference of the state-space model for *Paramecium*. The observed distance of *Paramecium* from the heat (first panel), the observed and inferred velocity of movement (second panel), the inferred coefficient of the heat (third panel), and the inferred random fluctuations of the velocity (last panel) are shown. **B** Estimation of the state-space model for *Paramecium* using the Kalman filter. The observed distance of *Paramecium* from the heat (first panel), the observed and inferred velocity of movement (second panel), and the inferred coefficient of the heat (third panel) are shown. **C** Estimation of the start time for *Paramecium* in without the state-space model. The observed distance of *Paramecium* from the heat is shown. In the panels including inferred values, dots, solid lines, and shaded regions are the observed values, medians, and 99 % confidence intervals, respectively. In **A**–**C**, orange solid lines represent the start time estimated by each method. The shaded and light regions are the periods without and with heat, respectively.
